# Efficacy and safety of intraoperative partial spray of 50% glucose for patients with spontaneous pneumothorax

**DOI:** 10.1186/s13019-024-02486-4

**Published:** 2024-01-20

**Authors:** Ryota Kiriyama, Shota Nakamura, Hironori Oyamatsu, Seijiro Niimi, Takaki Fujimura, Takehiko Okagawa, Toyofumi Fengshi Chen-Yoshikawa

**Affiliations:** 1 Department of Thoracic Surgery, Okazaki City Hospital, 3-1 Gosyoai, Koryuji-cho, Okazaki, 444-8553, Japan; 2 Department of Thoracic Surgery, Nagoya University Graduate School of Medicine, 65 Tsurumai-cho, Showa-ku, Nagoya, 466-8550 Japan

**Keywords:** 50% Glucose solution, Pneumothorax, Pleurodesis, Bullectomy, Video-assisted thoracic surgery (VATS)

## Abstract

**Objectives:**

The management for pneumothorax patients involves surgical intervention, nevertheless postoperative recurrences are often encountered. To reduce the rates of recurrence, thoracic surgeons have experimented with various novel techniques, such as pleural abrasion, chemical pleurodesis, and staple line coverage with absorbable sheets, in addition to bullectomy. And in recent years, there have been reports of the effectiveness of the use of intraoperative glucose intrapleural spray (GIS) containing 50 ml of 50% glucose solution in addition to bullectomy. However, information on the effects and adverse events of GIS is limited. Current study was aimed to assess the efficacy and safety of GIS in preventing recurrence of pneumothorax.

**Patients and methods:**

We conducted a retrospective study with 74 cases of bullectomy with or without GIS between 2018 and 2021 at Okazaki City Hospital. Of these cases, 50 received GIS (GIS group) while 24 were treated conservatively (C group).

**Result:**

The GIS group consisted of 46 males and 4 females, whereas the C group consisted of 23 males and 1 female, with mean ages of 38.5 ± 5.7 years and 30.5 ± 6.7 years, respectively. The GIS group exhibited a mean increase in blood glucose of 23.8 mg/dL postoperatively, and postoperative infections were observed in 2 cases in the GIS group (4.0%) and 2 cases in the C group (8.3%). The NRS scores of the patients in the GIS group and the C group three hours postoperatively were 4.0 and 3.1, respectively (*p* = 0.28). No prolongation of postoperative drainage period by GIS was observed (1.2 days and 1.4 days in the GIS and C groups, respectively). Postoperative recurrence occurred in two patients from the C group. The postoperative total drainage volumes were 341.8 ± 25.2 ml and 74.2 ± 25.5 ml in the GIS and C groups, respectively, showing a significant increase in drainage volume (*p* < 0.01). None of them presented dehydration-related symptoms.

**Conclusions:**

The use of intraoperative glucose intrapleural spray is effective and safe in terms of preventing recurrences and postoperative complications.

## Introduction

A pneumothorax is an abnormal collection of air in the pleural space between the lung and the chest wall [1–3]. Pneumothorax is caused by several factors. A primary spontaneous pneumothorax occurs without an apparent cause and in the absence of significant lung disease [4, 5]. Secondary pneumothorax occurs in the presence of existing lung disease, including chronic obstructive pulmonary disease, asthma, and malignant tumor [4, 5].

The treatment of pneumothorax aimed to eliminate air collection and prevent a recurrence. Conservative treatment with observation is the treatment of choice in small air collection in the pleural space, while air drainage is the second choice of treatment in large air collection [1, 2, 4]. Occasionally, surgical treatment is required in cases of unsuccessful or recurrent conventional drainage [1, 2, 4]. Bullectomy was recently performed in daily practice, with the lowest recurrence rate of approximately 1%, and is recommended in cases of permanent air leak or recurrent pneumothorax [1]. However, the higher recurrence rate has become a concern since the widespread use of video-assisted thoracoscopic surgery (VATS) in the 1990s [1, 6, 7]. The view of magnification is an advantage in the VATS procedure, nevertheless the narrow field of view is its disadvantage. The recurrence by VATS procedure is caused by the overlooking of bullae due to this narrow field and pulmonary collapse because of selective contralateral ventilation [8]. Therefore, reducing the recurrence rate by VATS is an urgent issue for thoracic surgeons. Recurrent pneumothorax is detrimental to young patients due to redrainage, reoperation, rehospitalization, and hospitalization costs. Thoracic surgeons have tried many novel techniques, such as partial pleurectomy, pleural abrasion, chemical pleurodesis, and staple line coverage with absorbable sheets, in addition to bullectomy, to reduce postoperative recurrence. Achieving a recurrence rate as minimal as feasible has been an enduring objective for surgeons, and if a novel technique presents itself with reduced complications and enhanced cost-effectiveness compared to the current approach, its implementation should be considered.

The efficacy of a glucose solution sprayed intraoperatively to prevent postoperative recurrence has been recently reported sporadically [7, 9–11]. However, information on the effects and adverse events of the intraoperative spray glucose solution is limited. This study aimed to retrospectively evaluate the efficacy and safety of this technique in patients with pneumothorax to prevent a recurrence.

## Patients and methods

### Ethical statement

This study was approved by the Institutional Review Board of Okazaki City Hospital in Japan (No. 2021-43). The informed consent requirement was waived because of the retrospective design of this study.

### Study design

**Fig. 1:**
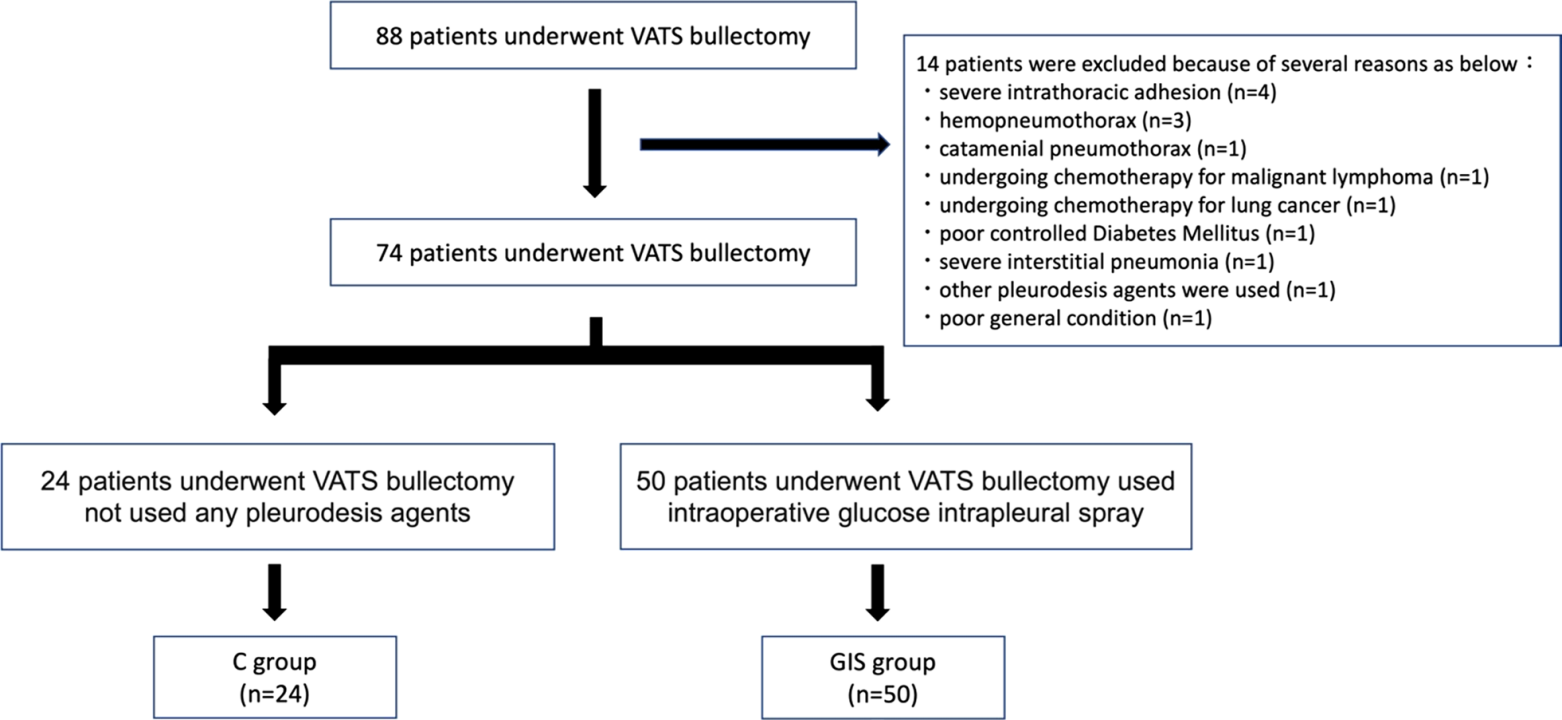
Details of the study enrollment. (VATS = video-assisted thoracoscopic surgery; C = conservative; GIS = Glucose solution intrathoracic spray)

This study retrieved 88 successive cases of surgical bullae excision from the thoracic surgical files of Okazaki City Hospital from 2018 to 2021. This study excluded 14 patients because of severe intrathoracic adhesion (n = 4), hemopneumothorax (n = 3), catamenial pneumothorax (n = 1), undergoing chemotherapy for malignant lymphoma (n = 1), undergoing chemotherapy for lung cancer (n = 1), poorly controlled diabetes mellitus (n = 1), severe interstitial pneumonia (n = 1), use of other pleurodesis agents (n = 1), and poor general condition (n = 1) (Fig. 1). Of the 74 patients, 24 underwent surgical intervention from April 2018 to July 2019, was not used intraoperative glucose intrathoracic spray (GIS), and were defined as the conservative (C) group. The other 50 patients underwent surgical treatment from August 2019 to November 2021, was used intraoperative GIS containing 50 ml of 50% glucose solution, and were defined as the GIS group. Postoperative complications were evaluated following the Clavien-Dindo classification [12].

### Endpoints

The endpoint of this study was evaluating for the postoperative results including postoperative recurrence, operating time, the total volume of chest tube drainage, the difference in blood glucose between pre- and postoperative levels, postoperative infections, the length of postoperative hospital stay, postoperative infection, pain intensity, and total duration with the drainage tube in place.

### Surgical procedures

**Fig. 2:**
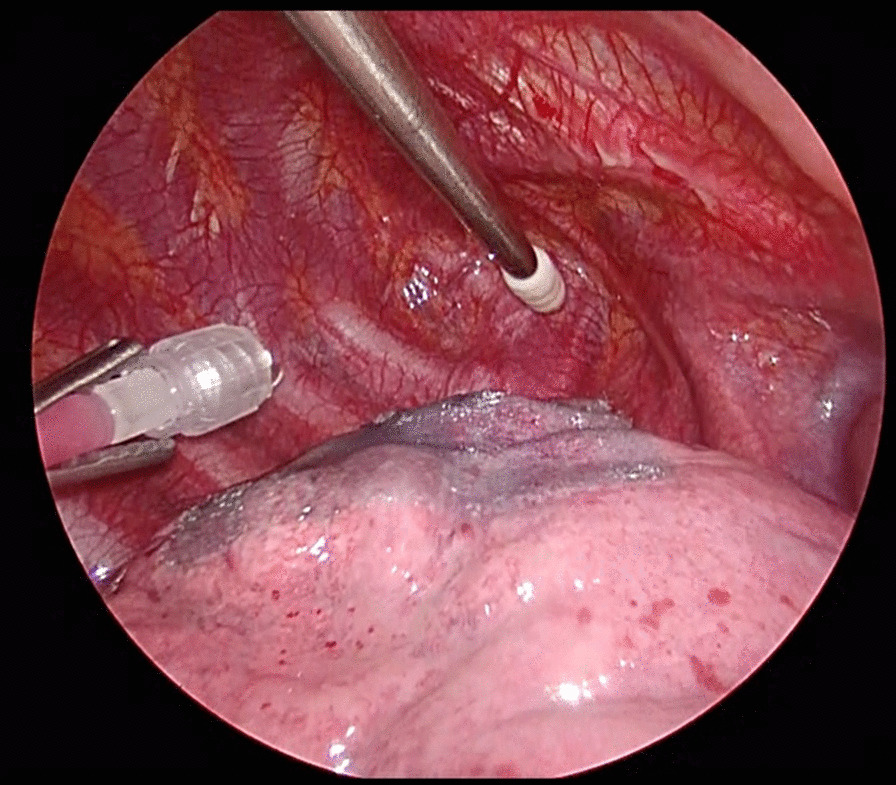
Intraoperative image spraying 50% glucose solution. We injected 50 ml of 50% glucose solution into the thoracic cavity using an intrathoracic catheter from an extrathoracic syringe

All patients underwent general anesthesia and were intubated with a double-lumen endotracheal tube to allow selective contralateral ventilation. Patients were placed in the lateral decubitus position. All procedures were performed by the VATS approach with a three-port, and the bullae were removed using an endoscopic linear stapler. A water sealing test at an airway pressure of 20 cmH_2_O was performed after the bullae resection to confirm the absence of air leakages, and a Polyglycolic Acid (PGA) sheet was applied on the staple line as coverage and reinforcement of the pleura. Additionally, the GIS group was sprayed 50 ml of 50% glucose solution mixed with 10 ml of 1% lidocaine hydrochloride on the lung surface around the stapled line or PGA sheet using a syringe and catheter (Fig. 2). Intercostal nerve block in all patients was performed with 0.375% ropivacaine for postoperative pain control. A 20-Fr thoracic drainage tube was placed in the intrathoracic cavity, the wound was closed, and the tube is connected to the continuous negative pressure suction device without any waiting time. Drain properties and drainage volume were recorded at 3, 6, and 24 h postoperatively. Additionally, the chest tube was removed if there were no air leakage or bloody effusion. Blood glucose levels were measured immediately and on the first postoperative day. Pain intensity was assessed at 3, 6, and 24 h postoperatively using the Numeric Rating Scale (NRS) [13]. Non-steroidal anti-inflammatory drugs and acetaminophen were used as analgesics regularly. The patient fasted from the morning of surgery day and resumed eating at noon 1 day postoperatively.

### Statistical analyses

All characteristics in the text and tables were expressed as the mean ± standard deviation. Welch’s t-test was used to analyze the total drainage volume. The Mann–Whitney U test was used to assess age, surgical duration, blood glucose level, pain intensity, postoperative drainage period, and length of postoperative hospital stay. Other data were evaluated by Fisher’s exact test. A *p* value of < 0.05 indicated statistical significance. Statistical analyses were performed using R statistical software version 4.1.2. statistical software.

## Result

### Patient characteristics

Patient characteristics are presented in Table 1. The GIS group consisted of 46 males and 4 females, whereas the C group consisted of 23 males and 1 female, with mean ages of 38.5 ± 5.7 years and 30.5 ± 6.7 years, respectively. Air leakage was observed in 32 (64.0%) and 14 patients (58.3%) in the GIS and C groups during surgery, respectively. Additionally, 22 (44.0%) and 6 (25.0%) patients in the GIS and C groups were seen in partial pleural adhesion, respectively (*p* = 0.13), and none were converted to open thoracotomy from the VATS procedure.

**Table 1:** Clinical characteristics of patients with pneumothorax who underwent video-assisted thoracic surgery, with or without glucose solution intrathoracic spray

Variable	GIS (n = 50)	C (n = 24)	*p* value
Age (years)	38.5 (32.8–44.3)	30.5 (23.8–37.3)	0.14
Sex			1.00
Male	46 (92.0%)	23 (95.8%)	
Female	4 (8.0%)	1 (4.2%)	
Smoking history			0.14
(−)	21 (42.0%)	15 (62.5%)	
(+)	29 (58.0%)	9 (37.5%)	
Classification of pneumothorax			0.57
PSP	37 (74.0%)	20 (83.3%)	
SSP	13 (26.0%)	4(16.7%)	
Intrathoracic adhesion			0.13
(−)	28 (56.0%)	18 (75.0%)	
(+)	22 (44.0%)	6 (25.0%)	
DM			1.00
(−)	48 (96.0%)	23 (95.8%)	
(+)	2 (4.0%)	1 (4.2%)	

Continuous data are presented as mean ± SD

*GIS* glucose solution intrathoracic spray, *C* conservative, *DM* diabetes mellitus, *PSP* primary spontaneous pneumothorax, *SSP* secondary spontaneous pneumothorax

### Surgical result

**Fig. 3:**
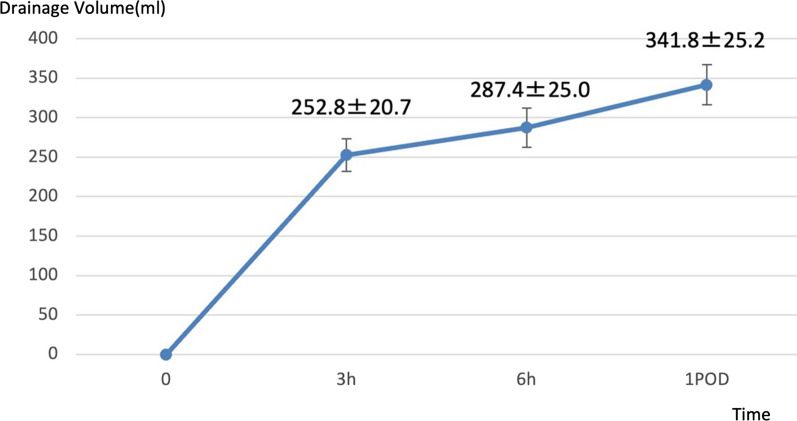
Postoperative drainage volume of the group given 50% glucose solution

Surgical results are presented in Table 2. Operating time was not significantly different between the GIS (82.6 ± 9.6 min) and the C groups (68.2 ± 12.9 min) (*p* = 0.07). The mean preoperative and postoperative blood glucose levels in the GIS group were 97.3 ± 3.8 mg/dL and in the C group were 121.5 ± 7.5 mg/dL, respectively, with a difference of 23.8 ± 7.4 mg/dL. The blood glucose level in the GIS group 1 day postoperatively was 100.1 ± 3.1 mg/dL, comparable to the preoperative level. Postoperative infections were observed in two cases each in the GIS (4.0%) and C groups (8.3%) (*p* = 0.59), all wound infections. Wound infections were grades I and II and grades I and IIIa in each case in the GIS and C groups, respectively. No significant difference was seen in the NRS scores between the GIS and C groups 3 h postoperatively (4.0 and 3.1 in the GIS and C groups, respectively) (*p* = 0.28). The NRS in the GIS group was 3.9 and 3.3 at 6 h and 1 day postoperatively, respectively, with no significant worsening of pain. Postoperative pulmonary leakage was observed in 5 and 3 patients in the GIS and C groups, respectively, with no significant difference (*p* = 0.71). No cases have postoperative pulmonary leakage of grade ≥ III. The total duration with the drainage tube in place was 1.2 ± 0.2 days and 1.4 ± 0.6 days in the GIS and C groups, respectively, with no significant difference. The length of postoperative hospital stay was 3.9 ± 0.4 days and 3.3 ± 0.6 days in the GIS and C groups, respectively. Postoperative recurrence was observed in two patients in the C group, and the period of postoperative recurrence was 4 and 6 months. The GIS group was followed up postoperatively for at least one year, and no recurrence was observed. The postoperative total drainage volumes were 341.8 ± 25.2 ml and 74.2 ± 25.5 ml in the GIS and C groups, respectively, showing a significant increase in drainage volume (*p* < 0.01). The postoperative drainage volume in the GIS group was > 200 ml 3 h postoperatively, afterward the drainage volume increased slowly (Fig. 3). The blood urea nitrogen/creatinine ratio of 5 (10.0%) patients in the GIS group was ≥ 25 on the first postoperative day. None of them presented dehydration-related symptoms.

**Table 2:** Surgical Results and advertise events of patients with pneumothorax who underwent video-assisted thoracic surgery, with or without glucose solution intrathoracic spray

Variable	GIS (n = 50)	C (n = 24)	*p* value
ΔBG (mg/dL)	23.8 ± 7.4	N/A	
Postoperative infection	2	2	0.59
Pain after 3 h on NRS	4.0	3.1	0.28
Pain after 6 h on NRS	3.9	N/A	
Pain after 1POD on NRS	3.3	N/A	
Total drainage volume (ml)	341.8 ± 25.2	74.2 ± 25.5	< 0.01
Postoperative drainage period (day)	1.2 ± 0.2	1.4 ± 0.6	0.58
Postoperative leakage	5	3	0.71
Recurrence	0	2	

Continuous data are presented as mean ± SD

NRS is shown as an average value

*GIS* glucose solution intrathoracic spray, *C* conservative, *BG* blood glucose, *ΔBG* pre- and post-operative blood glucose differences, *NRS* Numeric Rating Scale, *POD* post-operative day

## Discussion

We conducted a retrospective study concerning patients with pneumothorax and revealed GIS as effective and safe for preventing a recurrence. Partial pleurectomy or decortication is the conventional surgical procedure, considering surgical treatment for pneumothorax, and bullectomy may have been performed recently in daily practice [2, 4]. At present, surgical treatments for pneumothorax can be divided into three main components: resection of the bullae, suturing of the pulmonary fistula, and the creation of pleurodesis. The postoperative recurrence rate has ranged from approximately 5.0–10.0% [1, 14, 15]. Generally, the overlooking of bullae in surgical treatments, bullae regrowth, or bullae neogenesis are the common causes of postoperative recurrence of pneumothorax. Horio et al. revealed a significantly smaller number of resected bullae by VATS than by open thoracotomy, which is one of the reasons for the high recurrence rate with VATS [8].

Several surgical procedures have been reported to reduce the recurrence rate of postoperative pneumothorax. Absorbent sheet reinforcement is one of the methods for preventing recurrence [6, 14]. Additionally, pleurodesis is often performed intraoperatively using talc and has been reported as effective [16]. Lee et al. [6] conducted a prospective randomized controlled trial treating patients with pneumothorax and revealed a reduced recurrence rate with staple line reinforcement with fibrin glue combined with coverage of oxidized regenerated cellulose sheets. Cardillo et al. [16] reported that pleurodesis using talc poudrage reduced the recurrence rate to 1.73%. However, postoperative adverse events can occur by pleurodesis using talc which included acute respiratory distress syndrome (ARDS), respiratory failure, and difficulty in reoperation due to severe adhesions [1]. Recently, the efficacy of intraoperatively sprayed 50% glucose solution for partial pleurodesis to prevent postoperative recurrence has been reported sporadically [7, 9–11]. The European Respiratory Society task force describes the intrathoracic application of 50% glucose solution for primary spontaneous pneumothorax [2]. However, information on the specific clinical findings of glucose application is limited in intraoperative use.

GIS is expected to be effective in preventing a recurrence through two mechanisms. First, the osmotic pressure difference caused by the hypertonic glucose infusion stimulates and thickens the pleura; or pleural mesothelial cells and intrathoracic macrophages are stimulated to induce growth factors in the pleural fluid, which in turn stimulate inflammatory cells and fibroblasts, thereby reinforcing the pleura [7, 9, 11]. Second, the viscous nature of glucose enhances the adhesive effect of the PGA sheet, and the entire pleura, not just the sheet, is strengthened without boundaries, making the new bulla production difficult. The effectiveness of the intraoperative application of 50% glucose solution in preventing recurrence has been reported [7]. Tsuboshima et al. applied 50 ml of 50% glucose solution on an absorbable sheet intraoperatively to 106 of 376 patients with pneumothorax to prevent postoperative recurrence of spontaneous pneumothorax and analyzed the results. The results revealed smoking habit and glucose injection as factors in preventing pneumothorax recurrence, and they concluded that intraoperative application of glucose reduced the postoperative recurrence rate of spontaneous pneumothorax [7]. Our study revealed no recurrence in patients who underwent GIS. The recurrence rate for Group C was 8.3%, which was comparable to the previously reported recurrence rates. The recurrence observed in Group C suggests the expected efficacy, but this is one of the limitations of this study because of the retrospective selection bias.

Adverse events caused by 50% glucose solution were reported by several investigators. The adverse effects that should be noted when using 50% glucose solution include increased drainage volume, elevated blood glucose, increased pain [7, 9, 11], and even infection and respiratory failure. Tsukioka et al. injected 300–500 ml of 50% glucose in 18 cases and revealed bacterial pleuritis in 2 cases, describing the prolonged duration of drainage as a risk [9]. Some ARDS and respiratory failure cases have been reported in patients given 200–400 ml of 50% glucose solution [17]. However, the current study revealed no cases of major complications such as pneumonia, pleuritis, or respiratory failure. The reduced adverse effects may be related to the dosage of 50% glucose solution.

The dosage of 50% glucose solution varied from 50 to 500 ml depending on the literature, and our study used 50 ml. This is because a small amount of solution can be sprayed over the entire collapsed lung intraoperatively by endoscopically performing the procedure [16] and a small amount is expected to prevent the induction of massive pleural effusion and postoperative hyperglycemia.

Adverse effects of glucose pleurodesis include increased postoperative drainage volume and prolonged duration of the drain in place. The mean drainage volume was 605.7 ml according to Fujino et al. [11] and > 1000 ml in five out of seven cases according to Kajikawa et al. [10] in cases injected with 200 ml of 50% glucose solution. The mean volume of drainage was 337.4 ± 25.9 ml for our 50 ml dose, suggesting that the volume of drainage increases in proportion to the dose. Our study revealed no subjective symptoms and no cases of dehydration that could have led to complications although dehydration due to increased drainage should be noted when 50% glucose solution is administered [11]. Drainage volume after GIS revealed a slow increase at 3 h postoperatively and did not lead to a prolonged duration of the drain in place.

Moreover, hyperglycemia was cited as an adverse effect of pleurodesis with glucose. Fujino et al. [11] reported that 20 (43.5%) of 46 patients who received 200 ml of infusion had hyperglycemia of > 250 mg/dL. Additionally, Kajikawa et al. [10] reported hyperglycemia at > 200 mg/dL in four out of five cases. However, the current study reported only one case of hyperglycemia of > 200 mg/dL. Therefore, 50 ml of 50% glucose is an appropriate dose in terms of efficacy and prevention of complications.

Common pleurodesis agents, such as talc, cause pleural inflammation, and post-pleurodesis pain is a concern. Additionally, the pain has been noted as an adverse effect of glucose application. The 6-h postoperative NRS was 3.0 ± 2.2 [7], which was similar to our study, and with no significant difference between the GIS and C groups, and pain control was achieved with postoperative analgesics. However, it is important to note that our study does not provide a direct comparison with pleurodesis or partial pleurectomy, and the study design does not involve a prospective controlled or randomized controlled trial. Study results support the results gained by previous studies on the efficacy of GIS and may provide collateral evidence for its effectiveness. It is worth noting that the use of intraoperative glucose intrapleural spray may also represent a viable option for preventing recurrence following surgical treatment for patients with pneumothorax.

## Conclusion

In conclusion, this method uses inexpensive agents, is simple, and may be useful in terms of preventing complications. Prospective studies evaluating the efficacy of 50% glucose solution were not reported although several retrospective studies were reported on the efficacy of 50% glucose solution, and prospective studies showing the safety and efficacy of 50% glucose solution should be accumulated.

## Data Availability

The data underlying this article will be shared on reasonable request to the corresponding author.

## Abbreviations

VATS: Video-assisted thoracoscopic surgery
GIS: Glucose intrapleural spray
C: Conservative
PGA: Polyglycolic acid
NRS: The Numeric Rating Scale
ARDS: Acute respiratory distress syndrome
